# Trends in Reconstructive Hip Surgery for Cerebral Palsy in U.S. Community Hospitals Before and After Publication of National Hip Surveillance Guidelines

**DOI:** 10.1016/j.jposna.2026.100343

**Published:** 2026-02-18

**Authors:** Shana Kong, Jingyanshan Li, Shannon Tse, Miriam A. Nuño, Amanda T. Whitaker

**Affiliations:** 1 University of California Davis, School of Medicine, Sacramento, CA, USA; 2 Department of Orthopaedic Surgery, University of California Davis, Sacramento, CA, USA; 3 University of California Davis, School of Medicine, Division of Biostatistics, Sacramento, CA, USA; 4 Shriners Children's – Northern California, Sacramento, CA, USA

**Keywords:** Cerebral palsy, Hip surveillance, Hip subluxation, Hip displacement, Pelvic osteotomy, Femoral osteotomy

## Abstract

**Background:**

Hip surveillance guidelines have been introduced by the Australian (AusACPDM, 2008) and American Academy for Cerebral Palsy and Developmental Medicine (AACPDM, 2016) to screen for hip displacement in children with cerebral palsy (CP). Whether these guidelines are associated with changes in surgical management remains unknown. This study compares trends in hip osteotomy rates among children with CP in the United States using a national database before and after the publication of national hip surveillance guidelines.

**Methods:**

International Classification of Diseases (ICD-9)-CM and ICD-10-CM codes were used to identify hospital admissions for hip osteotomies in children <20 years old with CP from the Healthcare Cost and Utilization Project (HCUP) Kids’ Inpatient Database (KID) from 1997 to 2019. Cases without an admission month were excluded. National estimates of CP-related hospital cases, osteotomy (acetabular, proximal femoral) rates, and hip dislocations were calculated using weighted variables provided by HCUP. The average monthly osteotomy rates were compared using one-way analysis of variance (ANOVA) for the periods before and after guideline establishments. Baseline patient characteristics were also analyzed using either chi-squared tests or one-way ANOVA.

**Results:**

From 1997 to 2019, 318,367 weighted CP admissions were recorded, demonstrating a 44% increase in the annual incidence of CP hospitalizations. Baseline patient characteristics for the periods January 1997 to December 2006 (preAusACPDM), January 2009 to September 2016 (post-AusACPDM, pre-AACPDM), and October 2016 to December 2019 (post-AACPDM) indicates that mean age and length-of-stay (LOS) increased significantly in the post-AACPDM period relative to the two prior time periods (*P* < 0.01). The average monthly osteotomy rate was highest in the pre-AusACPDM period and decreased significantly beginning in the post-AusACPDM period, reaching its lowest level in the post-AACPDM period (*P* < 0.01).

**Conclusions:**

Reconstructive hip surgery rates declined among children with CP treated in U.S. community hospitals represented in the KID database, temporally coinciding with publication of national hip surveillance guidelines. These findings demonstrate an association rather than a causal relationship and may reflect variation in guideline adoption, shifts in care to tertiary referral centers, or evolving surgical practices.

***Key Concepts*:**

(1) Hip surveillance guidelines are screening tools for children with cerebral palsy.
(2) Early detection of hip subluxation is crucial for timely orthopaedic interventions.
(3) Children with cerebral palsy are undergoing hip osteotomies at older ages with longer stays in recent years.
(4) Barriers may exist that prevent adherence to hip surveillance guidelines.
(5) Formal guideline implementation may be necessary to improve timely interventions and outcomes.

**Level of Evidence:**

IV

## Introduction

Children with cerebral palsy (CP) are at significantly higher risk for hip subluxation due to an imbalance of muscular forces, abnormal bony development, and lack of weightbearing. Up to one-third of children with CP experience hip subluxation [[1], [2], [3], [4], [5]]. Progression to dislocation occurs in up to 20%, and is associated with further morbidity including pain, pressure ulcers, degenerative arthritis, and an overall reduction in quality of life [[6], [7], [8], [9]]. Surgical hip reconstruction via femoral and/or acetabular osteotomy is effective for improving hip stabilization, migration percentage, and hip pain in this population [[10], [11], [12]]. Early detection is important because timely orthopaedic intervention reduces the risk for dislocation, joint malformation, and less successful salvage surgeries [[13], [14], [15]].

Implementation of successful single site and regional hip surveillance programs in Australia and Sweden have been associated with decreased hip dislocation rates and mean age at time of hip surgery [6,[13], [14], [15], [16], [17]]. While hip dislocation has remained the primary outcome measure for hip surveillance efficacy, trends in reconstructive hip surgery rates such as femoral and pelvic osteotomy have also been studied as a secondary indicator of surveillance. Dobson et al. evaluated the impact of a hip surveillance clinic at a single tertiary center in Australia and found that during their three years of operation, the number of reconstructive surgeries increased, while the number of salvage surgeries decreased [14]. Haggard et al. conducted a twenty-year evaluation of South Sweden's regional hip surveillance program and found an increase in the number of reconstructive surgeries, as well as a decrease in both salvage surgeries and total repeat hip operations [16]. When including soft tissue release, Elkamil et al. found no significant difference in total hip operation rates between CP children enrolled in a hip surveillance program in South Sweden and those receiving regular care in Norway [6]. However, femoral osteotomy surgery rates were significantly higher in Sweden, where children were formally surveilled [6]. These studies highlight a consistent trend toward higher reconstructive surgery rates and lower age at surgery in CP children under strict surveillance.

In 2008, the first national radiographic hip surveillance guidelines were established by the Australian Academy of Cerebral Palsy and Developmental Medicine (AusACPDM) [16]. The Pediatric Orthopaedic Society of North America (POSNA) conducted a survey in 2014 in which 92% of surgeons reported that they would follow guidelines if established, and that 18% were already screening according to AusACPDM guidelines [18]. Subsequently, the American Academy of Cerebral Palsy and Developmental Medicine (AACPDM) developed their own national radiographic hip guidelines, which were first presented at the national AACPDM meeting September 2016. The AACPDM guidelines include a combined clinical examination with radiographic examination at varying intervals, depending on the patient's age, Gross Motor Function Classification System (GMFCS) level, and Winters, Gage, and Hick gait classification for early detection of hip displacement [19]. Most recently, the POSNA developed formal hip surveillance guidelines for children with CP in 2021 [20]. However, these guidelines were not included in the present analysis because their publication occurred after the final year of available data for this study.

To date, no study has evaluated national trends in reconstructive hip surgery in the United States in relation to the publication of the AusACPDM and AACPDM hip surveillance guidelines. Consequently, how the timing of these national surveillance recommendations relates to patterns of surgical management for neuromuscular hip dysplasia in the United States remains unclear.

This study aims to compare trends in hospitalization rates for reconstructive hip surgery in children with CP within the United States before and after the establishment of two national hip surveillance guidelines, given the absence of a formal surveillance program to directly assess the incidence of hip dislocation. We sought to evaluate whether national osteotomy rates changed following publication of each guideline.

## Methods

### Study design and data source

Hospital discharge data were extracted from the Kids’ Inpatient Database (KID), a pediatric-specific inpatient dataset within the Healthcare Cost and Utilization Project (HCUP), maintained by the Agency for Healthcare Research and Quality. The KID is the largest publicly available all-payer pediatric inpatient care database in the United States and includes a stratified sample of inpatient discharges from participating hospitals across multiple states. All hospitals contributing to the KID are fully de-identified, and individual institutions cannot be identified; therefore, it is not possible to determine which specific academic or tertiary pediatric referral centers are included or excluded. The database releases data every 3 years, and consists of monthly data from over 4,000 hospitals across the country, and includes patients under the age of 20. Datasets from 1997, 2000, 2003, 2006, 2009, 2012, 2016, and 2019 from the KID were available for analysis and were used for this study. The time periods of interest were January 1997 to December 2006, January 2009 to September 2016, and October 2016 to December 2019.

Qualifying medical records were identified using the International Classification of Diseases, Ninth Revision, Clinical Modification (ICD-9-CM) and the ICD-10-CM diagnosis and procedure codes. Discharge records were included in our study if they had the relevant diagnosis and procedure codes attached to the encounter. All discharges without an admission month were excluded. The HCUP provides reports of total counts of all ICD-9-CM and ICD-10-CM codes from 2000 to 2019 in their database, and these weighted totals were used to confirm that our study's data collection design was accurate. Comparable verification data were unavailable for the 1997 KID release; however, this dataset was included because it represents a full additional KID release, contributing unique observations from an earlier time point and extending the longitudinal scope of the analysis. The absence of ICD code–level verification data for this year is acknowledged as a limitation.

### Patient selection and exposure

All children <20 year old with a diagnosis of CP were included. ICD-9-CM 343.0, 343.1, 343.2, 343.3, 343.4, 343.8, 343.9 from 1997 to 2012 and ICD-10-CM diagnostic codes G80.0, G80.1, G80.2, G80.3, G80.4, G80.8, G80.9 from 2016 to 2019 were included.

From the CP population of interest, those who had pelvic or femoral osteotomies were identified. The ICD-9-CM procedure codes for osteotomies were 77.25, 77.29, and 77.35. This was correlated with the following 43 ICD-10-CM procedure codes used for 2016-2019: 0Q860ZZ, 0Q863ZZ, 0Q864ZZ, 0Q870ZZ, 0Q873ZZ, 0Q874ZZ, 0Q883ZZ, 0Q884ZZ, 0Q890ZZ, 0Q893ZZ, 0Q894ZZ, 0Q8B0ZZ, 0Q8B3ZZ, 0Q8B4ZZ, 0Q8C0ZZ, 0Q8C3ZZ, 0Q8C4ZZ, 08P830ZZ, 08P833ZZ, 08P834ZZ, 0P840ZZ, 0P843ZZ, 0P844ZZ, 0P8T0ZZ, 0P8T3ZZ, 0P8T4ZZ, 0P8V0ZZ, 0P8V3ZZ, 0P8V4ZZ, 0Q800ZZ, 0Q803ZZ, 0Q804ZZ, 0Q820ZZ, 0Q823ZZ, 0Q824ZZ, 0Q830ZZ, 0Q833ZZ, 0Q834ZZ, 0Q8Q0ZZ, 0Q8Q4ZZ, 0Q8R0ZZ, 0Q8R3ZZ, 0Q8R4ZZ.

The annual incidence of CP hospitalizations was reported per 1,000 total pediatric hospitalizations in each HCUP KID data file. Since hip surgery is a large and infrequent event, the rate was calculated as an average of monthly surgeries per 1,000 CP hospitalizations for each period. Average baseline patient characteristics, including age and sex, as well as hospital length of stay (LOS) were reported for each period.

### Statistical analysis

The discharge weight variable provided by HCUP was applied to estimate national pediatric discharges from all hospitals. The primary outcome - average monthly osteotomy surgical rates - was calculated and compared using one-way ANOVA with post-hoc Tukey-Kramer tests across three periods before and after the establishment of the AusACPDM and AACPDM guidelines: 1) January 1997 to December 2006 (preAusACPDM), 2) January 2009 to September 2016 (post-AusACPDM, pre-AACPDM), and 3) October 2016 to December 2019 (post-AACPDM). Descriptive statistics for baseline characteristics and LOS were reported and compared with either one-way ANOVA or chi-squared tests. All statistical analyses were performed using SPSS (IBM, Chicago, IL) with statistical significance at *P* < 0.05.

## Results

**Figure 1:**
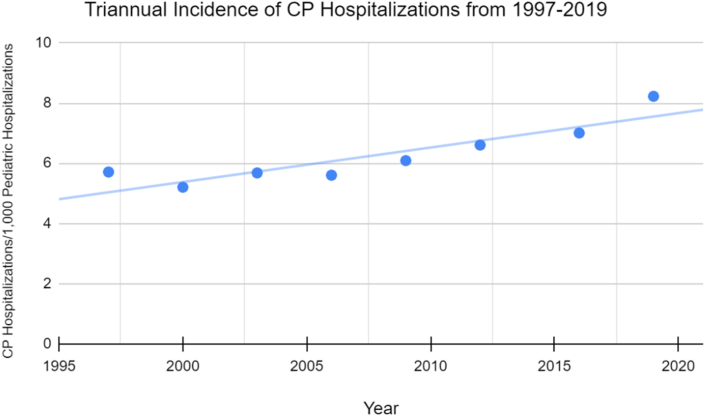
Triennial incidence of cerebral palsy hospitalizations from 1997 to 2019. The triennial incidence of CP hospitalizations increased by 44% across this period.

From 1997 to 2019, there were 318,367 weighted hospitalizations with an attached diagnosis of CP. The annual incidence of CP hospitalizations increased from 5.7 per 1,000 to 8.2 per 1,000 total pediatric hospitalizations annually (44%) across this period (Fig. 1).

When comparing baseline characteristics of patients between the three time periods, the mean age (*P* < 0.01) and LOS (*P* < 0.01) were significantly lower during the later time period 1 (Table 1). The gender distribution was majority male and comparable across all time periods (Table 1).

**Table 1:** Characteristics, LOS, and average monthly hip osteotomy rates for the time periods before and after the advent of AusACPDM and AACPDM guidelines in 2008, and September 2016, respectively.

	Time period 1: January 1997–December 2006	Time period 2: January 2009–September 2016	Time period 3: October 2016–December 2019	*P*-value
Weighted CP (N)	143,578	119,359	55,437	
Mean age	9 ± 5.64	10.16 ± 5.73	10.24 ± 5.72	<0.01
% males (n)	57.3 (69,792)	57.4 (68,487)	57.2 (31,732)	0.77
Mean LOS	5.95 ± 14.56	6.25 ± 11.79	7.01 ± 13.53	<0.01
Average monthly osteotomy rate (per 1,000 CP hospitalizations)	43	32	7.4	<0.01

*CP, cerebral palsy*; *LOS, length-of-stay*.

**Figure 2:**
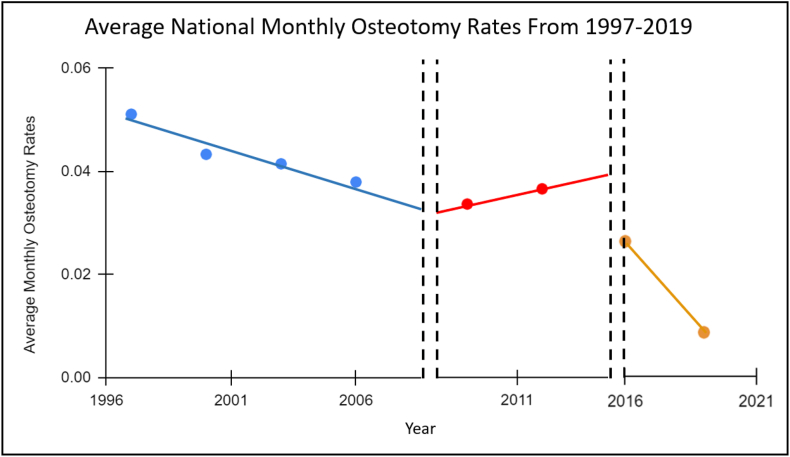
Trends in osteotomy rates from 1997 to 2019.

The average monthly osteotomy rate was the highest during period 1 at 43 surgeries per 1,000 CP cases, decreased to 32 per 1,000 in period 2, and decreased further in period 3 at 7.4 surgeries per 1,000 CP cases (*P* < 0.01) (Fig. 2).

The difference between the annual incidence of CP hospitalizations that we reported differed from the weighted totals provided in the documents on the HCUP website by 1.47–12.7%. This can be accounted for by the exclusion of all records without an admission month as this would limit accuracy in calculating average monthly surgery rates. This difference was accepted as accurate given the large sample size of the study.

## Discussion

To the best of our knowledge, this study is the first to evaluate national trends in reconstructive hip surgery in children with CP using a large, representative sample of the US population. Our study found that, despite an increase in CP–related hospitalizations in the United States, rates of reconstructive hip surgery decreased over the study period within community hospitals represented in the KID. Although this decline temporally coincided with the publication of the AusACPDM and AACPDM hip surveillance guidelines, a causal relationship cannot be established. These findings likely reflect a combination of factors including variability in guideline dissemination and adoption, shifts in care to tertiary pediatric referral centers not captured by this database, and evolving surgical practice patterns, rather than a direct effect of the guidelines themselves.

The Centers for Disease Control (CDC) reports the national prevalence of CP in the US to be 3 per 1,000 children, which remained stable from 1985 to 2002 [21]. Our study found a triennial incidence of CP hospitalizations that has increased to more than twice this rate. This further supports prior literature demonstrating that children with CP are at risk for frequent hospitalizations compared to non-CP pediatric peers [21,22]. Our study also showed a 44% increase in the triennial incidence of CP hospitalizations over the past 20 years. According to a recent regional population-based study, the prevalence of CP appears to have decreased over the past decade [23]. The increase in hospitalizations relative to a decreasing prevalence of CP indicates a potential overall increase in medical intervention for children with CP, either indicating a higher acuity patient population, more severe CP types, or increased survival among individuals with CP. Differentiating among these possibilities is not feasible within the KID dataset as patient-level measures of disease severity, including GMFCS level, are not available.

Another possible contributor to the observed decline in reconstructive hip surgery rates is the increased use of true preventative interventions such as adductor myotomy or guided growth, which may delay or reduce the need for subsequent bony reconstructive procedures. These interventions were not included in the present analysis as the study was designed to specifically evaluate trends in pelvic and proximal femoral osteotomies.

The mean age at the time of reconstructive hip surgery for children with CP in our study was approximately 9 years during 1997-2006, which increased to a mean of 10.2 years in 2016-2019. While this is higher than some studies that reported age at first surgery to be around 5 years, this is consistent with other studies demonstrating that older age was a predictor of osteotomy surgical success [6,14,[24], [25], [26]]. Furthermore, previous work has shown that effective hip surveillance is associated with earlier surgical intervention, resulting in younger age at reconstructive hip surgery [6,14], which was not demonstrated in our study after the establishment of the AusACPDM or AACPDM guidelines. The increase in mean age at surgery is likely multifactorial. One possible explanation is that patients are presenting with greater medical complexity, consistent with rising rates of CP-related hospitalizations and increasing LOS. Another contributing factor may be a deliberate delay in hip surgery to reduce the risk of future revisions [26,27]. Additionally, the increasing acceptance of single-event multilevel surgery (SEMLS) as the standard of care for CP patients who suffer from spasticity could also explain the increased mean age and CP-related hospitalizations [28]. The SEMLS is particularly advantageous in reducing the overall surgical burden and recovery time [29]. While multilevel surgery is ideally conducted between ages 6-12 [28], several studies report cohorts with mean ages of 9-12 years, reflecting variability in the timing of surgical intervention reported in the literature [30,31].

Contrary to our hypothesis, rates of reconstructive hip surgery significantly declined following the establishment of both the AusACPDM and AACPDM guidelines. While prior studies outside of the US have reported increased rates of reconstructive hip surgery in areas with formal hip surveillance programs [6,14], our study displays a consistent downtrend in hospitalization rates despite the introduction of the guidelines. A 2024 study by Chiu et al. utilizing a large all-payer claims database in the US, found increasing rates of reconstructive hip surgeries among CP patients under age 5 between 2010 and 2021, with a concurrent decrease among those aged 5 to 10 years old from 2016 to 2021, coinciding with the establishment of the AusACPDM and AACPDM guidelines [24]. One possible reason for the discrepancy between our findings and preexisting literature is that we did not investigate rates of soft tissue release surgery in addition to reconstructive bony hip surgery. While some studies have found an increase in both soft tissue release and hip osteotomies in patients being adequately surveilled [6,14], we excluded soft tissue releases. Soft-tissue procedures are often performed as true preventative interventions in selected patients with less severe hip displacement and have demonstrated efficacy in reducing progression to dislocation in prior studies [[32], [33], [34]]. However, these procedures were not included because the current study was designed to specifically evaluate trends in bony reconstructive pelvic and proximal femoral osteotomies, and administrative discharge data do not allow assessment of clinical or radiographic indications necessary to interpret soft-tissue procedures within a surveillance framework.

Another reason contributing to our differing results is that prior studies were conducted in countries with single-payer healthcare systems where formal hip surveillance programs have been successfully implemented with longitudinal follow-up [3,6,13,14,16,35]. These programs have their own CP-specific databases where surgery and the incidence of hip dislocation can be tracked. While organizations like the AACPDM and POSNA provide surveillance guidance in the US, implementation is left to individual care teams, leading to variations in adherence and monitoring practices. Notably, a cross-sectional study of multidisciplinary CP providers identified several barriers to hip surveillance implementation, including inconsistent radiologic reporting, parental disengagement, ambiguous assignment of responsibility, and inadequate communication among clinicians [36].

Moreover, CP care in the US is often centralized at large academic centers with specialized expertise, and most CP studies are also from these institutions; access to such facilities may be limited in certain regions [37,38]. While the HCUP KID database is useful for generating national estimates, it primarily includes data from community hospitals in more populous states, may exclude academic centers where many CP-related hip surgeries likely occur. Although resource availability and patient characteristics might differ between academic and community hospitals, care for children with CP, particularly complex reconstructive hip surgery, has increasingly become centralized at large tertiary pediatric referral centers in the United States. Since the KID database primarily captures inpatient discharges from community hospitals and does not permit identification of individual institutions, trends observed in this study may not reflect surgical practices at these tertiary centers. To date, no studies have directly compared hip surveillance practices or reconstructive surgical interventions across hospitals with differing resource levels in the United States [25]. This limitation underscores the challenges of both implementing and evaluating national hip surveillance recommendations in the absence of a centralized hip surveillance program or CP–specific registry.

The primary strength of this study is its use of a large, multisite, nationally representative dataset. However, there are some limitations. The KID captures hospital discharges rather than individual patients, preventing longitudinal analysis or patient-level assessment of disease severity, additional or salvage surgeries, or GMFCS levels. Hospitals contributing to the KID are fully de-identified, and hospital characteristics are reported only in aggregate; as a result, it is not possible to determine the average hospital size or the number of academic pediatric centers represented. Since the KID primarily reflects inpatient discharges from community hospitals, reconstructive hip surgeries performed at large tertiary pediatric referral centers may be underrepresented, and observed trends may reflect shifts in where care is delivered rather than true national changes in surgical frequency. Additionally, data in the KID only provide information on prevalence of hip dislocations, which are a chronic condition once they occur in children with CP. The transition from ICD-9-CM to ICD-10-CM in 2015 may have introduced coding inconsistencies. Finally, limited data from the KID after 2016 restricts the ability to fully assess the long-term impact of the AACPDM guidelines.

In conclusion, rates of reconstructive hip surgery for the treatment of neuromuscular hip dysplasia decreased over time in U.S. community hospitals represented in the KID, despite an increasing number of CP–related hospitalizations. The average monthly osteotomy rate declined significantly from 1997 to 2019. While this decline temporally coincided with the publication of national hip surveillance guidelines from the AusACPDM and AACPDM, a causal relationship cannot be determined from this study. Mean age at surgery and hospital length of stay increased over the study period; however, these findings should be interpreted cautiously as this analysis cannot assess surgical indications, disease severity, or patient-level outcomes. As additional longitudinal data become available, future studies may better clarify the relationship between hip surveillance practices, timing of reconstructive surgery, and long-term hip outcomes in children with CP.

## Ethics approval and consent

The author(s) declare that no patient consent was necessary as no images or identifying information are included in the article.

## Author contributions

**Shana Kong:** Writing – review & editing, Writing – original draft, Visualization, Validation, Project administration, Methodology, Investigation, Formal analysis, Data curation. **Jingyanshan Li:** Writing – review & editing, Writing – original draft, Visualization, Validation, Investigation, Formal analysis. **Shannon Tse:** Writing – review & editing, Writing – original draft, Visualization, Validation, Supervision, Methodology, Formal analysis. **Miriam A. Nuño:** Writing – review & editing, Validation, Supervision, Software, Methodology, Formal analysis, Data curation, Conceptualization. **Amanda T. Whitaker:** Writing – review & editing, Writing – original draft, Visualization, Supervision, Software, Resources, Methodology, Investigation, Funding acquisition, Formal analysis, Data curation, Conceptualization.

## Funding

We had no funding for this project.

## Declaration of competing interests

The authors declare the following financial interests/personal relationships which may be considered as potential competing interests:

Amanda Whitaker reports a relationship with Pacira BioSciences Inc that includes: consulting or advisory. Amanda Whitaker reports a relationship with UptoDate Inc that includes: consulting or advisory. Amanda Whitaker reports a relationship with National Institutes of Health that includes: funding grants. Amanda Whitaker reports a relationship with Pediatric Orthopaedic Society of North America that includes: funding grants. Amanda Whitaker reports a relationship with Commission for Motion Laboratory Accredidation that includes: board membership. None If there are other authors, they declare that they have no known competing financial interests or personal relationships that could have appeared to influence the work reported in this paper.
